# Book Review

**Published:** 1894-03

**Authors:** 


*On Diseases of the Lungs and Pleuræ, including Consumption.* By R. DOUGLAS POWELL, M.D. Fourth Edition. Pp. ix., 600. London: H. K. LEWIS. 1893.

The third edition of this book was published in 1886. No material change in the plan of the work has now been made, but the etiology, pathology, and treatment of consumption have necessarily received a large measure of revision: the author has strenuously endeavoured to withdraw attention from the too exclusively microbic view which the public are coming to hold, and he has endeavoured to point out how easily due measures of prophylaxis may be taken without creating pain or causing needless separation in families. He emphasises the importance of adopting every possible measure of cleanliness with regard to the disposal of the expectoration from phthisical patients, and he recommends destruction by fire as by far the best and easiest way of dealing with the sputum, which in private cases can be simply poured on to the fire, and in hospitals can be placed in some kind of furnace.

Spittoons containing liquids of a more or less destructive character are in common use in the belief that sputum is only dangerous when dry; but it is far better to do away with the necessity for cleaning these receptacles by adopting the device of the New York Sanitary Supply Company, whose spitting-cup consists of a metal frame, into which fits a stiff paper case, which is easily taken out and thrown into the fire. For out-of-door use, patients are urged to carry Dettweiler's pocket spittoons, which are flask-shaped, and can be easily carried in the pocket. The abomination is the dried handkerchief: these should never be allowed to become dry, but if used at all must be changed frequently, well disinfected, and scalded before going to the wash with other things. These and other precautions must be adopted in the interest of the patient himself, who is the most susceptible to unhygienic surroundings, and is most exposed to the danger of setting up fresh centres of disease by the inhalation of bacillary dust.

The *British Medical Journal* has recently (Sept. 16th, 1893) made enquiry as to the rules adopted at various hospitals, has given a full report thereon, and urges "a consistent and methodical warfare against infectious dust, for which purpose we must insist on the use of the spittoon, the substitution of pieces of paper or of calico, which can be burned, for that horrible thing, the consumptive's handkerchief, the abolition of ornaments and knick-knacks in the sick man's room, and the replacement of the duster by the damp cloth." (*B. M. J.*, Sept. 23rd, p. 696.)

Much reiteration will be needful before these precautions are generally adopted: people will not believe that they are necessary, and will only carry them out when they are insisted on by the medical attendant. Until they are persistently enforced, dried bacillary dust must be as ubiquitous as the disease itself; but if we can "entrap the vehicle of infection, isolation may become as unnecessary as it now appears impracticable."

In the treatment of pneumonia we think that phenacetin deserves a more prominent position than is given to a drug which not only is a more efficient antipyretic than the five-grain doses of quinine which the author advises, but is also a useful anodyne and an efficient diaphoretic. In the absence of any indication for more active interference, a five-grain dose of phenacetin may be given frequently, every three or four hours, and no other treatment may be needful; this will be likely to remove the necessity for the cold pack or the cool bath, and we have never seen any untoward symptoms from its use, even when given in five-grain doses every hour until the temperature had fallen to a safe level.

The author advises small doses of digitalis in various conditions where there may be symptoms of heart-failure, but he makes no allusion to the modern use of large doses of this drug in the way advised by Petresco and others. (See *Bristol*

*Medico-Chirurgical Journal*, Vol. x., p. 196.) Neither does he mention the utility of chloral hydrate in cases of active delirium, where a few doses of this drug give an exhausted patient a restful sleep, which must favour the maintenance of heart-power, even when given without the digitalis in the combination so strongly recommended by Balfour (*Ed. Med. Journ.*, Nov., 1891). We hold that a rapidly fatal disease with so high a mortality as pneumonia is one which calls for active combat, and in which a venturesome policy is not only justifiable but is urgently needed.

The author sums up the treatment of pleural effusion in a series of excellent rules (p. 113), only one of which do we see any sufficient reason for criticising. The last of them contains the following: "Provided the pus be perfectly sweet, it is, I think, best not at once to operate upon a large effusion by making a free opening. The lung is compressed, and it is better in the first instance to remove a portion of the fluid by means of the syphon, for the double purpose of re-expanding the lung texture and diminishing the cavity of the abscess to be subsequently (within a short time, however) dealt with." We fail to see any necessity for this delay, and have never had reason to regret the practice of free incision as soon as the diagnosis of empyema has been established.

The book, like its author, has a well-founded reputation; it has proved itself to be a necessity to any medical library and a friend to those who consult it.

*What to Do in Cases of Poisoning*. By William Murrell, M.D. Seventh Edition. Pp. 276. London: H. K. Lewis. 1893.—This little book is justly popular with the Resident Medical Officers of Hospitals, and unquestionably serves a very useful purpose. But having reached a seventh edition, it should have had the mistakes of earlier issues carefully corrected. We were surprised to find conspicuous errors of grammar and spelling: *e.g.*, pp. 38, 157. More serious is the oversight on p. 131, where for "four minims of laudanum" should be read "one-fourth of a minim."

*Mathews' Medical Quarterly: A Journal devoted to Diseases of the Rectum, Gastro-Intestinal Disease, and Rectal and Gastro-Intestinal Surgery*. Joseph M. Mathews, M.D., Editor and Proprietor. Louisville: John P. Morton & Company.—We have received the first number of this new quarterly medical journal, which we are informed is the only journal of the kind published. It contains no less than 190 pages of reading matter, with nineteen original contributions from well-known surgeons in America. The printing and paper are of the usual Transatlantic excellence, and if forthcoming numbers are up to the standard of this one, it will reflect the highest credit upon editor and publisher. In this country we cannot quite understand the necessity of specializing to the extent now adopted in America. We presume from one or two paragraphs in this journal that there are numerous surgeons in American cities who devote their practice solely to diseases of the rectum! We can only look on with wonder and astonishment, and ask what will be the next development.

*The Year-Book of Treatment for 1894.* London: Cassell and Company, Limited.—We welcome the early appearance of this useful Annual, now too well known to need introduction to medical practitioners. Its scope and general arrangement differ in few respects from the issues of preceding years, but we are glad to note that it has followed the example of the *Medical Annual* by introducing two much needed sections; viz., the medical diseases of children, undertaken by Dr. Dawson Williams, and bacteriology, which has been entrusted to Dr. William Hunter: both these names are sufficient guarantee that these sections are done well. We note with pleasure that Dr. E. Markham Skerritt and Dr. Barclay Baron continue to be responsible for the same sections as heretofore. The general arrangement of the work and the editing as a whole leave little to be desired, but a defective index and the absence of cross-references are faults that might be remedied with advantage. Thus salipyrin is not included among the new remedies; it has two lines devoted to it as an anti-rheumatic remedy, but it does not appear at all in the index.

*Transactions of the New York Academy of Medicine.* Vol. VIII. Pp. xxviii., 393. 1892.—This volume is full of most interesting and valuable papers. It opens with one by Dr. A. Jacobi on "Cervical Adenitis: its Etiology, Symptoms, and Diagnosis." He comments much upon the statement that "there is no better protection to the system than intact integument;" that cracked lips, every form of stomatitis, the cracked tongue of typhoid, and so on, may be "an inlet to micrococci of all sorts," and that it is this which destroys more patients than the original typhoid infection. An interesting point raised by Dr. Wm. H. Thompson was the question, "Why children should have adenitis nine times to adults once." A paper by Dr. Max Einhorn introduces a new deglutable electrode for direct faradization of the stomach. Dr. H. G. Piffard, on "Lupus," remarks "that we need not, then, be surprised that the lupus yields in a measure to the repeated pyrexiæ induced by the Koch injections." As regards the use of tuberculin in pulmonary tuberculosis, after much evidence from many sources had been adduced, the president, Dr. Alfred L. Loomis, "thought the consensus of opinion was that we had in tuberculin an agent powerful for good and evil, and that it would take a permanent place in the treatment of pulmonary tuberculosis." Dr. R. W. Taylor discusses the question, "Why syphilis is not aborted by excision of its initial lesion with a liberal slice of the surrounding parts." The pathology of the eclampsia and albuminuria of pregnancy is discussed by Dr. R. Van Santvoord, and the medical and surgical treatment of epilepsy by Dr. B. Sachs. The volume contains an interesting and excellent obituary memoir of Dr. Fordyce Barker, with many other original and valuable communications.

*A Practical Text-book of the Diseases of Women.* By ARTHUR H. N. LEWERS, M.D. Fourth Edition. Pp. xvi., 439. London: H. K. LEWIS. 1893.—The best evidence of the popularity of Dr. Lewers' book is the rapid appearance of its fourth edition. The author does not introduce much new matter, nor materially alter that which has appeared in previous editions. He now limits the use of solid dilators for the exploration of the uterine cavity to those cases in which dilatation is required shortly after parturition or abortion, which is perhaps rather too narrow a field for their use. This edition is interesting, as lapse of time has enabled the author to follow up and publish the condition of several patients on whom he had performed the operation of supra-vaginal amputation of the cervix uteri, and renders still more instructive the tables of cases which he introduced into his earlier editions, which show very clearly the advantages of performing the above operation in the way he advises. The general arrangement of the book, the thorough and excellent instructions given for case-taking, with its clearness and attention to detail throughout, cannot fail to render it one of the best hand-books of the subject at present published, and particularly valuable to the student of gynæcology.

*A Practical Treatise on Materia Medica and Therapeutics.* By JOHN V. SHOEMAKER, M.D. Second Edition. Vol. I. Pp. xi., 354. Vol. II. Pp. vi., 355—1046. Philadelphia: The F. A. DAVIS COMPANY. 1893.—Within two years from the date of publication of the first edition, a second has been called for of this truly encyclopædic work. Due advantage has been taken to bring the information on materia medica and methods of treatment up to date in a singularly complete manner, such novelties as chloralose, alumnol, and thyroid extract being treated of in their proper places. As in the previous edition, the first volume is devoted to the consideration of remedial agents not generally classed as drugs, such as electricity, heat and cold, while the second volume is devoted to drugs proper. The drugs are arranged in the alphabetical order of their names, irrespective of their origin or affinity, so that, as in a dictionary, any substance may be found at once without trouble; each volume also is separately and elaborately indexed. We cannot speak too highly of this work, which in its way is far in advance of anything published in this country.

*Tooth Extraction.* By JOHN GORHAM. Fourth Edition. Pp. xvi., 43. London: H. K. LEWIS. 1893.—This is a most disappointing booklet. By its title and handy size it promises to be useful to the general medical student, and it is quite large enough to convey the few hints which a novice in extraction needs before he actually starts to perfect himself by practice. But it is misleading. Its tables, professedly to enable the tyro to distinguish between a temporary and permanent tooth, must lead to disaster: Nature—at least, human nature—may conform to generalisations, but not to rules. It is out of date; for it refers to the use of the key, as if this barbarous instrument were to be advocated: nowadays it should not even be tolerated. The book is also dangerous; for the canons which forbid the use of the elevator in the upper jaw, especially for upper wisdoms, were not established without reason. While unnecessarily prolix in some parts—*e.g.*, on the lever forces—the work is wretchedly meagre in others—*e.g.*, on anæsthetics. In so small a book there seems no particular necessity for the introduction of this latter subject; but, being introduced, it should not be summarily dealt with.

*The Johns Hopkins Hospital Reports.* Vol. III. Nos. 4, 5, 6. Report in Pathology, III. Baltimore: THE JOHNS HOPKINS PRESS. 1893.—The volume now issued contains four articles detailing observations on pathological anatomy, one on the histology of the cerebellar cortex, and one on the action of bacteria on plants. Of the former the most important is, perhaps, that by Dr. Simon Flexner on "Multiple Lymphosarcoma or Hodgkin's Disease," in which the modern tendency to regard this disease as due to the action of micro-organisms is supported by fresh observations and arguments. Dr. H. L. Russell's paper, on "Bacteria in their Relation to Vegetable Tissue," is an account of an elaborate experimental investigation of the results of inoculation of plants with various species of micro-organisms, both pathogenic and non-pathogenic.

*Diet Charts.* London: H. K. LEWIS.—In these we have a suggestive set of diet tables for handing to patients after consultation. The set includes albuminuria, alcoholism, anæmia and debility, constipation, diabetes, diarrhœa, dyspepsia, fevers, gout, nervous diseases, obesity, phthisis, and rheumatism (chronic). The charts are likely to save much time when full details in the regulation of diet are needed. Such details may not often be essential, but in cases of diabetes, chronic dyspepsia, and obesity they cannot be dispensed with; and these charts, compiled with care, embody the views of the best authors on the respective subjects.

*Disinfectants and Antiseptics: How to Use Them.* By EDWARD T. WILSON, M.B. 25th thousand. London: H. K. LEWIS.—Dr. Wilson's little hand-card contains in a small space very useful hints for householders in the presence of infectious sickness. These cards deserve a wide circulation.

*Ambulance Card.* By LLEWELLYN MORGAN, M.D. Liverpool: WINSTANLEY AND WATKINS.—This is intended for the use of ambulance classes. It is a handy table of the symptoms of the different forms of insensibility.

